# Aesthetic impact of resin infiltration and its mechanical effect on ceramic bonding for white spot lesions

**DOI:** 10.1186/s12903-024-04011-4

**Published:** 2024-03-21

**Authors:** Jiaen Shu, Yijia Huang, Xueying Ma, Zhonghua Duan, Pei Wu, Sijing Chu, Yuqiong Wu, Yuhua Wang

**Affiliations:** 1 Department of Prosthodontics, Shanghai Ninth People’s Hospital, Shanghai Jiao Tong University School of Medicine, 200011 Shanghai, China; 2 College of Stomatology, Shanghai Jiao Tong University, Shanghai, China; 3 National Center for Stomatology, Shanghai, China; 4 National Clinical Research Center for Oral Diseases, Shanghai, China; 5 Shanghai Key Laboratory of Stomatology, Shanghai, China; 6 Shanghai Research Institute of Stomatology, Shanghai, China

**Keywords:** White spot lesions, Resin infiltration, In-office bleaching, Bonding properties, Surface properties

## Abstract

**Background:**

Treating white spot lesions (WSLs) with resin infiltration alone may not be sufficient, raising questions about its compatibility with other treatments amid controversial or incomplete data. Therefore, this study aimed to assess the aesthetic feasibility of resin infiltration combined with bleaching, as well as its potential mechanical effect on ceramic bonding to WSLs.

**Methods:**

One hundred and fifty flat enamel surfaces of bovine incisors were prepared. Ninety specimens were deminerailized and randomly assigned to three groups(*n* = 30): post-bleaching resin infiltration (Bl-R), pre-bleaching resin infiltration (R-Bl), and only resin infiltration (R). Color, surface roughness and microhardness were assessed in immediate, thermocycling and pigmentation tests. The remaining sixty samples were randomly assigned to three groups (*n* = 20): control (Ctrl), bonding (Bo), pre-bonding resin infiltration (R-Bo). Shear bonding strength, failure mode, micro-leakage depth and interface morphology were evaluated after ceramic bonding. The Tukey test and analysis of variance (ANOVA) were used for statistical analysis.

**Results:**

For the effect of resin infiltration and bleaching on WSLs, the R-Bl group showed the worst chromic masking ability, with the highest |ΔL|, |Δa|, |Δb|, and ΔE values after treatment. Compared with those in the Bl-R group, the R-Bl and R groups showed significant time-dependent staining, which is possibly attributed to their surface roughness. For the effect of resin infiltration on the adhesive properties of WSLs, resin infiltration reduced the staining penetration depth of WSLs from 2393.54 ± 1118.86 μm to 188.46 ± 89.96 μm (*P* < 0.05) while reducing WSLs porosity in SEM observation.

**Conclusions:**

Post-bleaching resin infiltration proved to be advantageous in the aesthetic treatment of WSLs. Resin infiltration did not compromise bonding strength but it did reduce microleakage and enhance marginal sealing. Overall, resin infiltration can effectively enhance the chromatic results of treated WSLs and prevent long-term bonding failure between ceramics and enamel. Based on these findings, the use of post-bleaching resin infiltration is recommended, and resin infiltration before ceramic bonding is deemed viable in clinical practice.

**Supplementary Information:**

The online version contains supplementary material available at 10.1186/s12903-024-04011-4.

## Background

White spot lesions (WSLs) typically appear when changes in pH levels cause the enamel matrix to dissolve internally. This process results in a porous enamel structure, leading to the formation of opaque patches on the tooth surface [1]. These lesions are often seen in the early stages of enamel caries [2], dental fluorosis [3], enamel developmental defects [4], and during orthodontic treatment with fixed appliances [5]. The prevalence of WSLs varies from 2 to 97% due to various causal factors [5–7]. However, they are commonly observed in adolescents as a result of inadequate oral hygiene and extensive orthodontic treatments [5]. Some researches indicate no significant gender differences in the occurrence of WSLs [8], while others report a two to three times higher incidence in males within orthodontic populations [9]. Nevertheless, certain researchers observe a higher prevalence in females [10]. Treatment options for WSLs include microabrasion [11, 12], external bleaching [11, 13], remineralization [14–16], resin infiltration [17–22], and ceramic veneer restoration [3, 23]. Among these options, resin infiltration, a minimally invasive approach, has emerged as a “gold standard” intervention method.

Resin infiltration, primarily consisting of Triethylene Glycol Dimethacrylate, exhibits low viscosity, a reduced contact angle, and a high penetration coefficient. This composition facilitates effective penetration into demineralized enamel, resulting in the formation of a resin-matrix complex during the curing process [22]. Both clinical and in-vitro studies have established its significant role in enhancing the aesthetic and mechanical properties of enamel affected by WSLs [17–20]. Aesthetically, resin infiltration is praised for its ability to replicate the natural translucency, reflectance, and fluorescence of healthy enamel [24, 25]. Mechanically, it has been demonstrated to elevate surface micro-hardness, reduce surface roughness, and enhance resistance to acidic challenges [26, 27]. However, it is essential to acknowledge that in cases of severe fluorosis or structural degradation, resin infiltration alone may not be adequate to achieve the desired aesthetic results [11, 21, 28, 29], while bleaching and veneer restoration is reserved for situations where infiltration falls short [3]. Understanding the interactions between resin infiltration and subsequent procedures is essential for treatment compatibility.

In-office external bleaching, often employing hydrogen peroxide [24] or carbamide peroxide [13, 30] at varying concentrations, is a common procedure frequently combined with resin infiltration for managing WSLs [13, 24]. Bleaching effectively conceals discolorations by enhancing the overall lightness of enamel and reducing its yellowness [24]. However, bleaching has the potential to compromise the hardness of WSLs enamel by causing additional mineral loss [31]. Pre-bleaching resin infiltration has demonstrated effectiveness in mitigating the reduction in tooth hardness following bleaching [11, 28, 32, 33]. Furthermore, post-bleaching resin infiltration has proven effective in eliminating surface stains and reducing yellowish discoloration, leading to improved teeth whitening outcomes and greater patient satisfaction, as substantiated by various in vitro and clinical studies [11, 28, 32, 33]. Nevertheless, uncertainty persists regarding the optimal treatment sequence and the relative efficacy of combined treatments, due to divergent findings concerning chromatic recovery and durability [24, 28, 33], as well as a lack of data on long-term color stability after exposure to common staining agents.

When minimally invasive methods fail, adhesive restoration becomes a viable alternative. Concerns regarding the potential impact of resin infiltration on bonding processes persist, especially when it penetrates up to 900 μm into WSLs [34], exceeding the typical enamel removal depth of 300–500 μm for veneer restorations [35]. Additionally, limited data is available on the influence of resin infiltration on the bonding performance of WSLs and ceramics in vitro.

This paper aimed to evaluate the aesthetic effect of resin infiltration and bleaching on WSLs and the impact of resin infiltration on the ceramic adhesive properties of WSLs. The first null hypothesis (H0) was that there would be no significant difference in the short-term or long-term effect of chromatic recovery of WSLs when treated with post-bleaching resin infiltration, pre-bleaching resin infiltration, or resin infiltration alone. The second null hypothesis (H0) was that there would be no significant difference in the shear bonding strength, failure pattern, or microleakage depth between WSLs treated with or without resin infiltration.

## Materials and methods

This research project (Ref. number: SH9H-2022-TK148-1) was approved by the research ethics committee of the Shanghai Ninth Hospital in accordance with the ethical guidelines of the Helsinki Declaration as revised in 2000.

### Specimens collection and preparation

One hundred and fifty bovine incisors from a slaughterhouse were prepared by removing the pulp using a low-speed handpiece and burs, cleaning the pulp cavities, and filling with wax. Subsequently, the teeth were embedded in acrylic resin. The enamel surfaces were polished using 800, 1000, 1200, and 2400 grit silicon carbide paper. A 6 × 6 mm^2^ section of enamel surface on each specimen was retained for future use, while the surrounding enamel was coated with two layers of color-free, acid-resistant nail varnish (Nail Enamel, Revlon, Oxford, England) to ensure precise dimensions [14].

The sample size was calculated using G*Power software (3.0.10, Christian-Albrechts-Universitat, Kiel, Germany) based on the results based on a pilot study. Nine teeth per group in each experiment were required to have an 99% chance of detecting as significant at the 5% level (2-sided test), with a minimum detectable difference in means of 5.35 and expected standard deviation of 3.8. To compensate possible losses, the sample size was added 10%, resulting in 10 teeth per group [36].

To induce the formation of WSLs on the labial surfaces of the enamel, the specimens were immersed in a demineralization solution (2.2 mM CaCl_2_, 2.2 mM NaHPO_4_, 50 mM acetic acid, pH adjusted to 4.4 with 1 M NaOH) for 96 h at 37 °C, with daily changes to the solution [37, 38].

### Experimental design of the study

**Fig. 1:**
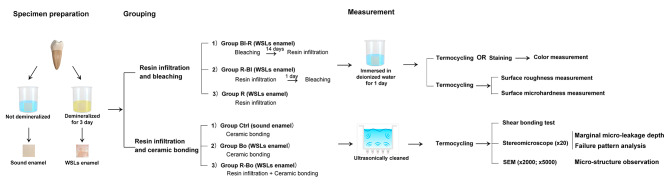
Study design

The study design and the materials used are presented in Fig. 1; Table 1.

The impact of resin infiltration on bleaching was analyzed in 3 WSL groups at different treatment stages (*n* = 30): Group Bl-R (post-bleaching resin infiltration), Group R-Bl (pre-bleaching resin infiltration), and Group R (resin infiltration alone).

The mechanical effect of resin infiltration on ceramic bonding were analyzed in 3 groups (*n* = 20): the control (Ctrl), Bo (bonding), and R-Bo (pre-bonding resin infiltration) groups.

**Table 1:** Materials used

	Type	Composition	Manufacturer	Lot number
Icon-Etch	Etching agent	15% hydrochloric acid	DMG, Hamburg, Germany	231,816 250,846
Icon-Dry	Drying agent	100% Ethanol	DMG, Hamburg, Germany	231,823
Icon-Infiltrant	Infiltrant agent	BisGMA, TEGDMA, Ethanol, Camphorquinone, DABE	DMG, Hamburg, Germany	218,736 231,811
Opalescence Boost in-office whitening	Tooth whitening system	40% hydrogen peroxide, potassium nitrate, fluoride	Ultradent, South Jordan, America	BKJTS
Eco-Etch	Etching agent	37% phosphoric acid	Ivoclar Vivadent, Schaan, Liechtenstein	Z04BZ4
IPS Ceramic Etching gel	Etching agent	hydrofluoric acid	Ivoclar Vivadent, Schaan, Liechtenstein	Z02DGZ
Monobond Plus	Silane coupling agent	Tetrabutylammonium hydrogen fluoride, methacrylic acid phosphate, 3-(trimethoxysilyl)propyl acrylate, 1,2-bis(triethoxysilyl)ethane, water, 2-butan Alcohol, 1,3-butanediol and Fast Green	Ivoclar Vivadent, Schaan, Liechtenstein	Z03V76
Syntac Primer	Bonding agent	TEGDMA, polyethylene glycol dimethacrylate, maleic acid, acetone	Ivoclar Vivadent, Schaan, Liechtenstein	Z03VHR
Syntac Adhesive	Bonding agent	polyethylene glycol dimethacrylate, glutaral	Ivoclar Vivadent, Schaan, Liechtenstein	Z04TVR
Heliobond	Bonding agent	Bis-GMA, TEGDMA	Ivoclar Vivadent, Schaan, Liechtenstein	Z00RM3
Variolink N	Dual-cure bonding agent	BisGMA, urethane dimethacrylate, TEGDMA, barium glass, ytterbium trifluoride, Ba-Al-fluorosilicate glass, and spheroid mixed oxide	Ivoclar Vivadent, Schaan, Liechtenstein	Z02BLH
IPS e.max ZirCAD	Ceramics	Zirconium oxide, aluminum oxide, yttrium oxide, hafnium oxide	Ivoclar Vivadent, Schaan, Liechtenstein	YB54FJ Z01TC9

BisGMA, bisphenol A glycerolate dimethacrylate; TEGDMA, triethylene glycol dimethacrylate; DABE, ethyl 4-(dimelhylaminolbenzoate)

### Resin infiltration application

Using the Icon Treatment Set (Vestibular, DMG, Hamburg, Germany), Icon-Etch (15% hydrochloric acid) was applied for 2 min using the provided kit tips. It was left in place for 3 min, then rinsed for 30 s and dried with oil-and-water-free air. Icon-Dry (ethanol) was injected and held for 30 s before air-drying. Subsequently, Icon-infiltrant was applied to the surface, and any excess infiltrant was removed after being absorbed for 3 min. The teeth were subjected to a 40-s light curing process (Bluephase G4 [high power mode], Ivoclar Vivadent, Schaan, Liechtenstein) using a tip with a diameter of 4 mm, an intensity of 1200 mW/cm^2^, and a distance of 4 mm. The distance was maintained consistent with a customized stand for the light curing device. The Infiltrant was once again applied to the surface for 1 min and then subjected to another 40-s light curing process. Following completion, the specimen was immersed in deionized water for 1 day before measurement [33]. All materials were applied according to their manufactures’ instructions.

### In-office bleaching treatment

An Opalescence Teeth Whitening System (Boost in-office Whitening, Ultradent, South Jordan, America) with 40% hydrogen peroxide was used in the bleaching treatment. The Opalescence Boost was mixed, and the delivery head was installed. Bleaching gel was applied evenly to a thickness of 0.5–1 mm, held for 20 min, and removed; this process was repeated twice. After being rinsed for 1 min, the specimen was immersed in deionized water for 1 day before measurement [33]. All materials were applied according to their manufactures’ instructions.

### Thermocycling test

After demineralization, ten specimens from each group of specimens were placed in a thermocycler (TC-501; Weier, Suzhou, China) with a temperature control setting of 55 °C on one side and 5 °C on the other. The dwell time was 15 s, and the transition time was 5 s. A total of 5000 cycles were performed to simulate one year of tooth aging [32].

### Staining test

After treatment, twenty specimens from each group of specimens were randomized and immersed in cola (*n* = 10) or red wine (*n* = 10) for 4 h daily for 7 days to mimic anti-exogenous recoloration [20]. Color measurement was conducted on 0, 3 and 7 days after staining.

### Color measurement

The specimens were positioned on a spectrophotometer (MetaVue VS3200, X-Rite, Grand Rapids, America) for color comparison at different time points: T0 (baseline), T1 (after demineralization), T2 (after treatment), T3 (after thermocycling), as well as 0, 3 and 7 days after staining. A built-in calibration white board was utilized for color correction, and Color iQC (v10, X-Rite, Grand Rapids, America) was employed to analyze the L* (Luminosity, lightness), a* (red-green axis), and b* (yellow-blue axis) values using the International Illumination Commission (Commission Internationale de l’Eclairage, CIE) detection method [39]. Each specimen underwent three repeated measurements. The overall color difference ΔE was calculated by using $\varDelta \text{E}={\left[{\left({\text{L}}_{Tx}-{\text{L}}_{\text{T}0}\right)}^{2}+{\left({\text{a}}_{\text{T}\text{x}}-{\text{a}}_{\text{T}0}\right)}^{2}+{\left({\text{b}}_{\text{T}\text{x}}-{\text{b}}_{\text{T}0}\right)}^{2}\right]}^{1/2}$. For each L*, a*, and b* value, the absolute value of the color difference was calculated, for instance, $|\varDelta {L}^{*}|=|{L}_{Tx}-{L}_{T0}|$.

### Surface roughness measurement

The specimens were tested on a contact surface roughness meter (MAHR M1, Dailyaid, Beijing, China) at T0, T1, T2, and T3. All the specimens were measured three times, and the arithmetic mean (Ra) was calculated.

### Surface microhardness measurement

The specimens were tested on a surface microhardness meter (HXD-1000TMC, Taiming, Shanghai, China) at T0, T1, T2, and T3. The test surface was positioned parallel to the ground, while the loading head maintained perpendicular alignment. The indenter was pressed against the specimen with a 25 g load for 15 s [40]. Three points were randomly selected, with the condition that the distance between any two spots should not exceed 50 μm. The mean value of the three tested points was recorded.

### Fabrication of ceramic blocks

Ceramic blocks (IPS e.max CAD C14, Ivoclar Vivadent, Schaan, Liechtenstein) were cut into dimensions of 5 × 5 mm^2^ with a thickness of 2 mm (± 0.01 mm) [41]. After sintering, the ceramic blocks were polished using silicon carbide abrasive paper with grit sizes of 800, 1000, 1200, and 2400. They were subsequently cleaned in an ultrasonic bath (SN-QX-13, Pushang, Shanghai, China) for 5 min to remove surface contaminants [42].

### Bonding procedures

The ceramic blocks underwent the following bonding procedures: Etching with 37% hydrofluoric acid (IPS Ceramic Etching gel, Ivoclar Vivadent, Schaan, Liechtenstein) for 2 min. Rinsing and ultrasonic cleaning (SN-QX-13, Pushang, Shanghai, China) for 3 min. Coating with a silane coupling agent (Monobond Plus, Ivoclar Vivadent, Schaan, Liechtenstein) followed by drying with oil-free air. Etching of the enamel from Group Ctrl, Bo and R-Bo with a gel containing 37% phosphoric acid (Eco-Etch, Ivoclar Vivadent, Schaan, Liechtenstein) for 20 s, followed by a 10-s wash and gentle air-drying for 10 s. Application of primer (Syntac Primer, Ivoclar Vivadent, Schaan, Liechtenstein) to the enamel for 15 s. Application of a bonding agent (Syntac adhesive, Ivoclar Vivadent, Schaan, Liechtenstein) for 10 s, followed by another bonding agent (Heliobond, Ivoclar Vivadent, Schaan, Liechtenstein) for 10 s. Bonding of blocks and specimens using dual-curing resin adhesive (Variolink N, Ivoclar Vivadent, Schaan, Liechtenstein), pressed with a 500 g weight and cured with light irradiation for 20 s on each side. All materials were applied according to their manufactures’ instructions. After bonding, all specimens underwent thermocycling as detailed in Sect. Thermocycling test.

### Shear bonding test

Ten bonding specimens were tested on a universal testing machine (EZ20, LLOYD, Fareham, England). A knife-edged attachment was used, positioned parallel to the bonding interface and as close as possible to the bonding surface. The initial force of the head was 0.5 N, and the rate of descent until failure was 0.5 mm/min [43]. The maximum load F upon failure was recorded, and the shear bonding strength P was determined by dividing F by S, where S was the bonding area (25 mm^2^).

### Failure pattern analysis

After completion of the shear bonding strength test, the debonded areas were observed under a stereomicroscope (×20) (Discovery. V12, Zeiss, Oberkochen, Germany) [44]. Failure patterns were classified into three categories [44, 45]:

1. Adhesive failure: The bond breaks inside the teeth or ceramic.
2. Cohesive failure: The bond breaks between the ceramic and the adhesive or between the body and the adhesive.
3. Mixed failure: Both cohesive and cohesive failure occurred.

### Marginal microleakage depth

Five specimens were randomly selected from Group Ctrl, Bo, and R-Bo. They were placed in a 2% methylene blue solution at 37 °C for 24 h [46]. Afterward, they were rinsed under running water, subjected to ultrasonic cleaning (SN-QX-13, Pushang, Shanghai, China) for 1 min, dried using ethanol, and embedded in acrylic resin. The specimens were then horizontally sectioned in a mesio-distal direction using an automatic micro-cutting machine (MECATOME T215, Pressi, Servon, France), resulting in three sections (each 1.5 mm thick) obtained from each specimen. The extent of methylene blue dye penetration between enamel and ceramic blocks was examined using a stereomicroscope (×20) (Discovery.V12, Zeiss, Oberkochen, Germany), and the length of dye leakage was recorded.

### SEM observation

Five specimens were randomly chosen from Group Ctrl, Bo, and R-Bo for electron microscopy at magnifications of ×2000 and ×5000 (Mira 3 XH, Tescan, Brno, Czech) [47]. These specimens, each sliced to a thickness of 1 mm with an automatic micro-cutting machine (MECATOME T215, Pressi, Servon, France),, were ultrasonically cleaned for 1 min and dried using ethanol. A 5 nm-thick gold nanoparticle thin film was evenly sprayed onto the specimens (Q150T ES PLUS, Quorum, East Sussex, England). The cross-section was then examined, and representative regions were selected for observation.

### Statistical analysis

GraphPad Prism (9.0, GraphPad Software, Santiago, America) was used to process the data. The data were presented as the mean ± standard deviation (SD). The Tukey test and analysis of variance (ANOVA) were used for comparisons with the following parameters: significance level: 0.05; confidence interval: 95%; power: 80%. NS indicates no statistical significance; *, **, and *** indicate that there were statistically significant differences.

## Results

### Modeling

Compared with those of healthy enamel (T0), the WSL enamel (T1) exhibited notable differences in color (Fig. S1A, Fig. S1B, Fig. S1C), surface roughness (Fig. S2A) and microhardness (Fig. S2B), which was consistent with the findings of previous studies [21, 28] and confirmed the efficacy of our WSL modeling method.

### Effects of resin infiltration and bleaching on WSLs

The aesthetic goal of WSLs is to ensure that the affected area visually appears similar to the surrounding healthy enamel. Visual color dissimilarities are reflected by the L*, a*, and b* values, as well as their color differences (ΔE) [48]. If ΔE<3.7, the color difference is thought to be imperceptible to human eyes [48].

All three groups of treated enamel (T2) exhibited masking effect, with |ΔL|, |Δa |, |Δb|, and ΔE values all lower than those of WSL enamel (T1) (Fig. S1A, Fig. S1B, Fig. S1C, Fig. S1D), which implied decreased color deviation from baseline (T0). Overall, the R-Bl group presented the highest |ΔL_T2_| (5.626), |Δa _T2_| (1.094) and |Δb _T2_| (3.775) values and the highest ΔE_T2_ (7.212), indicating the worst masking ability (Table 2). The R group presented the lowest |ΔL _T2_| (0.06) and |Δb _T2_| (0.578) (Table 2). The Bl-R group presented the lowest |Δa _T2_| (0.006), as well as the lowest ΔE _T2_ (5.829) (Table 2). Additionally, the Bl-R group had significantly greater L* values (*P* < 0.05) and lower b* values (*P* < 0.05) at T2 compared to T0 (Fig. S1A, Fig. S1C), indicating greater brightness and lower yellowness, providing a camouflage effect that made the whiteness of the lesion less visible [13] and potentially improving patient satisfaction.

Color stability should also be taken into consideration. Previous studies have measured material aging [49] and exposure to coloring agents [33] in the oral environment when assessing aesthetic outcomes. Before (T2) and after (T3) thermocycling, the average ΔE value only slightly changed in all three groups (Table 2; Fig. S1D). However, cola staining had a significant time-dependent impact on the R-Bl group (*P* < 0.05) and the R group (*P* < 0.05) (Table 3; Fig. S1E; Fig. S1F). The susceptibility to staining might be explained by surface properties [50]. The R-Bl group had the highest surface roughness (0.653 ± 0.087 μm) at T2, followed by the R group (0.487 ± 0.118 μm) and the Bl-R group (0.453 ± 0.105 μm) (Table 4; Fig. S2A). Consequently, the Bl-R group was proven to have the best durability.

**Table 2:** Differences in chromatic changes (△L, △a, △b, △E) of WSLs between the various groups (*n* = 10)

Time		R-Bl	Bl-R	R	ANOVA comparison		Multiple comparison		
							**R-Bl vs. Bl-R**	**R-Bl vs. R**	**Bl-R vs. R**
		**Mean ± SD**			**F Value#**	***P Value#***	***P Value¶***		
ΔL	T0 to T2	5.626 ± 3.703	2.150 ± 2.296	-0.060 ± 6.763	2.036	0.1556	0.2503	0.1408	0.7275
	T0 to T3	2.258 ± 3.730	-0.901 ± 5.596	0.940 ± 4.530	4.851	*	0.3176	0.8037	0.7049
Δa	T0 to T2	-1.049 ± 1.236	0.006 ± 1.147	-0.116 ± 0.921	2.165	0.1396	0.2155	0.2382	0.9699
	T0 to T3	-1.380 ± 1.084	0.103 ± 0.520	0.346 ± 0.659	11.150	*	*	*	0.6965
Δb	T0 to T2	-3.775 ± 1.450	-3.274 ± 2.230	-0.578 ± 1.786	6.821	*	0.8594	*	0.0466
	T0 to T3	-4.339 ± 1.553	-4.345 ± 2.209	-0.980 ± 1.907	8.276	*	&gt; 0.9999	*	*
ΔE	T0 to T2	7.212 ± 3.528	5.829 ± 2.261	6.143 ± 2.654	0.513	0.6062	0.6305	0.7763	0.9649
	T0 to T3	6.096 ± 2.132	6.024 ± 2.704	4.515 ± 1.881	1.242	0.3092	0.9981	0.2898	0.4231

T0 (baseline), T1 (after demineralization), T2 (after treatment), T3 (after thermocycling)

#ANOVA;¶Tukey’s multiple comparisons test;

Level of significance level at <0.05

**Table 3:** Differences in chromatic visibility of WSLs (△E) between the various groups after staining (*n* = 10)

Staining time		R-Bl	Bl-R	R	ANOVA comparison		Multiple comparison		
							**R-Bl vs. Bl-R**	**R-Bl vs. R**	**Bl-R vs. R**
		**Mean ± SD**			**F Value#**	***P Value#***	***P Value¶***		
Cola	Day 0	7.970 ± 3.939	7.160 ± 3.266	4.350 ± 2.184	2.800	0.0836	0.8962	0.1024	0.1482
	Day 3	16.102 ± 4.115	7.861 ± 2.958	8.550 ± 4.688	10.530	*	*	*	0.9347
	Day 7	18.981 ± 6.213	10.034 ± 5.313	10.762 ± 5.732	5.944	*	*	*	0.9626
Red wine	Day 0	7.317 ± 2.997	7.342 ± 2.857	4.733 ± 2.358	2.374	0.1176	0.9998	0.1727	0.1521
	Day 3	16.948 ± 4.846	13.139 ± 4.044	11.364 ± 3.927	3.536	*	0.2382	0.0597	0.6548
	Day 7	17.682 ± 5.852	14.970 ± 2.677	12.258 ± 4.929	2.686	0.0915	0.4847	0.1486	0.3905

#ANOVA;¶Tukey’s multiple comparisons test;

Level of significance level at < 0.05

**Table 4:** Differences in surface roughness and surface microhardness between the various groups (*n* = 10)

Time		R-Bl	Bl-R	R	ANOVA comparison		Multiple comparison		
							**R-Bl vs. Bl-R**	**R-Bl vs. R**	**Bl-R vs. R**
		**Mean ± SD**			**F Value#**	***P Value#***	***P Value¶***		
Surface roughness	T1	1.479 ± 0.295	1.436 ± 0.294	1.432 ± 0.381	0.063	0.9388	0.9895	0.9645	0.9924
	T2	0.653 ± 0.087	0.453 ± 0.105	0.487 ± 0.118	10.42	*	*	*	0.5594
	T3	0.703 ± 0.112	0.582 ± 0.150	0.631 ± 0.109	2.355	0.1141	*	*	0.9983
Surface microhardness	T1	51.040 ± 3.079	52.960 ± 8.549	54.590 ± 3.379	1.008	0.3783	0.8632	0.8375	0.9986
	T2	204.880 ± 44.102	246.290 ± 34.699	260.570 ± 18.642	7.179	*	*	0.1171	0.9099
	T3	215.910 ± 46.203	251.830 ± 36.053	251.051 ± 18.198	3.354	0.0500	0.313	0.66	0.8247

T0 (baseline), T1 (after demineralization), T2 (after treatment), T3 (after thermocycling)

#ANOVA;¶Tukey’s multiple comparisons test;

Level of significance level at < 0.05

In addition to aesthetic considerations, microhardness was measured. Vickers hardness analysis revealed that the R group (260.570 ± 18.642 VHN) (*P* > 0.05) and Bl-R (*P* < 0.05) group (246.290 ± 34.699 VHN) exhibited greater microhardness than the R-Bl group (204.880 ± 44.102 VHN) at T2 (Table 4; Fig. S2B), while no significant difference was observed among the three groups at T3 (*P* > 0.05) (Table 4).

### Mechanical effect of resin infiltration and ceramic bonding bleaching on WSLs

**Fig. 2:**
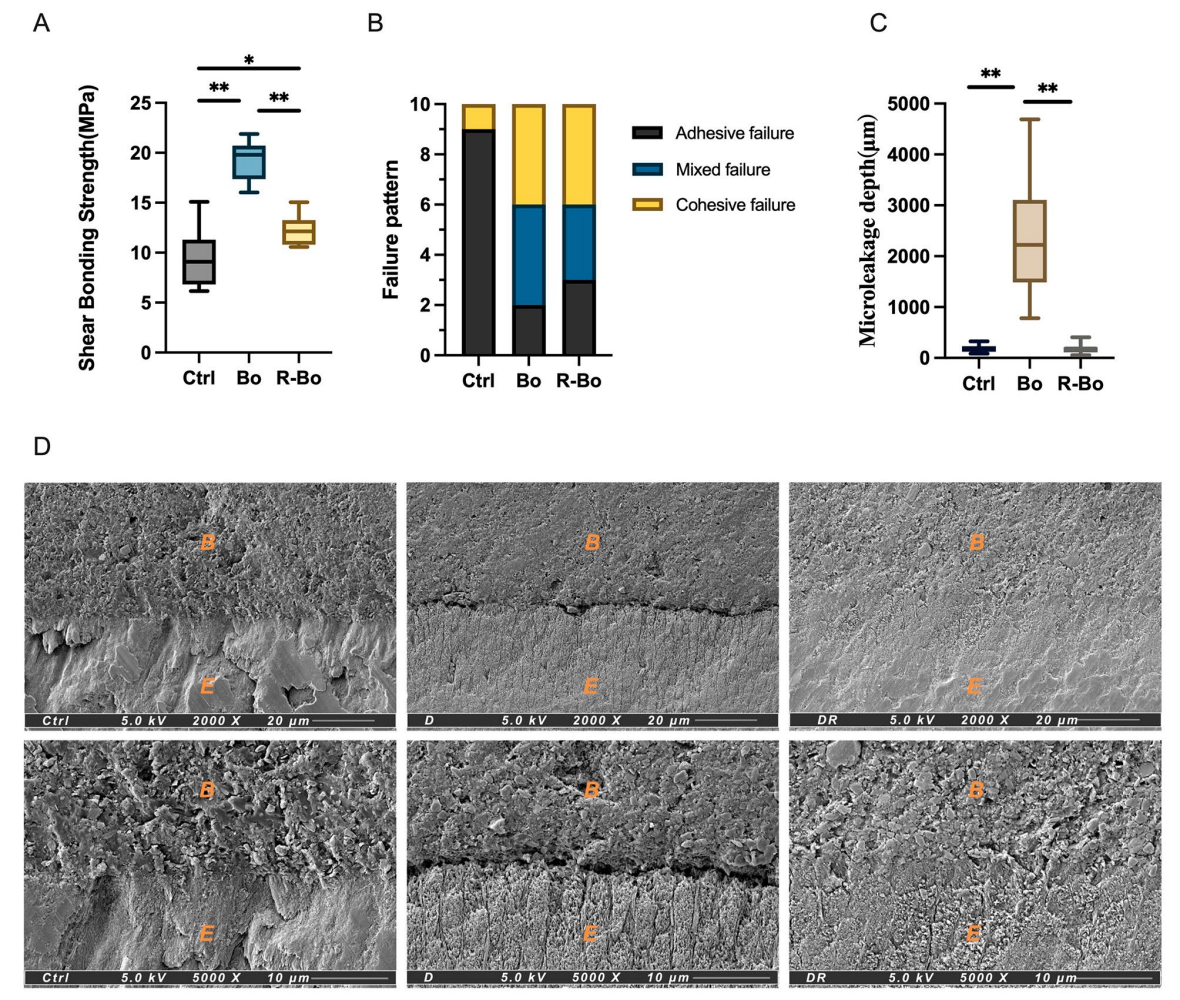
The effect of resin infiltration on the adhesive properties of WSLs. **(A)** The shear bonding strength of the Ctrl group, Bo group, and R-Bo group (*n* = 10). **(B)** Failure pattern analysis of the Ctrl group, D group, and DR group (*n* = 10). **(C)** The microleakage depth of the Ctrl group, Bo group, and R-Bo group (*n* = 15). **(D)** Representative SEM images of the Ctrl group, D group, and DR group (2000×, scale bar = 20 μm; 5000×, scale bar = 10 μm). The B zone: bonding agent; the E zone: enamel. *P < 0.01, **P < 0.001

Teeth preparation cannot eliminate the infiltrant in the enamel affected by WSLs, which may raise concerns about the treatment of resin-infiltrated teeth with ceramic veneers, as the quality of bonding is crucial. To evaluate changes in mechanical bonding properties following resin infiltration, shear bonding values were measured for three groups: Ctrl group (sound enamel), Bo group, and R-Bo group. The shear bonding strength (SBS) of the R-Bo group (19.18 ± 1.97 MPa) was significantly higher than that of the Ctrl group (9.57 ± 2.89 MPa) (*P* < 0.05), but it was significantly lower than that of the Bo group (12.26 ± 1.55 MPa) (*P* < 0.05) (Fig. 2A). This difference may be explained by the porous structure of the WSLs assisting in the formation of mechanical interlock, thus affecting bonding [51]. Nevertheless, resin infiltration did not decrease the shear bonding strength compared to that of sound enamel. In terms of failure patterns, the R-Bo group better mimicked the sound enamel than the Bo group(Fig. 2B). Like in previous studies [52], we found that sound enamel was prone to adhesive failure (90%), while those of the R-Bo group and the Bo group were 30% and 20%, respectively.

The failure to achieve tight marginal sealing is a primary cause of ceramic veneer failure [53, 54]. The micro-structure of the bonding interface was analyzed through micro-leakage testing and SEM observation. In the R-Bo group, the average length of the methylene blue micro-leakage line was 188.46 ± 93.12 μm, which was close to that of the Ctrl group (177.53 ± 71.36) (*P* > 0.05), but significantly lower than that of the Bo group (2393.54 ± 1158.13) (*P* < 0.05) (Fig. 2C). Electron microscopy observations further revealed a marginal closure in the R-Bo group that resembled sound enamel and exhibited zero pores or vacuoles between the enamel-ceramic interface. This indicates better marginal sealing compared to the porous and discontinuous Bo group, which is advantageous for the long-term prevention of caries and debonding [23, 54].

## Discussion

In this in vitro study, we assessed the aesthetic and mechanical ramifications of resin infiltration on demineralized bovine teeth. The demineralization protocol adhered to the methodology outlined by Kumar et al. [38] and resulted in a chromatic increase in the L* value and a decrease in the a* and b* values, as well as, mechanically, an increase in surface roughness and a decrease in microhardness. These findings verify the efficacy of the WSL model [28]. The use of human teeth of variable age and environmental exposure, as well as curved surfaces, presents challenges for achieving uniform treatment effect. Bovine teeth, which are flat and relatively consistent, serve as a reliable substitute due to their close chemical similarity to human teeth, particularly in comparison to porcine and ovine teeth [55]. They exhibit similar Ca/P ratios after remineralization and demineralization processes [56]. Bovine and human teeth also show no significant differences in mineral loss, lesion depth, or biofilm formation under cariogenic condition [57, 58], nor in the analysis of failure patterns. This supports the widespread adoption of bovine teeth in bonding evaluations [59, 60].

Remineralization, alongside resin infiltration, is a frequently employed, minimally invasive approach for treating WSLs [61]. This method utilizes various agents, including fluoride, nano-hydroxyapatite, bioactive glass, CPP-ACP, and modified infiltrative resins [62, 63], enhancing enamel strength and reducing tooth sensitivity [64], especially in teeth that are bleached or about to be bleached [65]. However, the effectiveness of this treatment depends on patient adherence, and WSLs may continue to exist despite thorough oral hygiene and diet management [66]. Clinical studies have shown that resin infiltration is more effective in halting caries progression than fluoride varnish in both primary and permanent teeth [67–69]. Additionally, a meta-analysis points out that the positive camouflaging effects of fluoride varnish cannot compete to resin infiltration, meanwhile might take up to six months, in contrast to the immediate masking effect of resin infiltration [70].

Thus, resin infiltration has been introduced to stop the advancement of non-cavitated lesions, conceal demineralized lesions, and preserve the natural translucency of enamel [71]. It can fully obscure the opaque color of inactive WSLs and partly conceal the appearance of moderate to severe WSLs reaching deep dentine [72]. The commercial ICON system (DMG, Hamburg, Germany) uses a three-step method: Icon-Etch (15% hydrochloric acid) for removing the superficial uneven layer, Icon-Dry (ethanol) to reduce the viscosity of the infiltrant and remove residual moisture, and Icon-Infiltrant to permeate porosities through capillary action [1].

Regarding modeling, acid etching was used to replicate the chromatic and mechanical characteristics typical of WSLs [37]. To investigate the impact of resin infiltration on bleaching, three groups of WSL enamel were treated with post-bleaching resin infiltration, pre-bleaching resin infiltration, or resin infiltration alone. Each specimen was immersed in deionized water for 24 h to remove any residual substances, in line with the evidence suggesting that storage times ranging from 24 h to 14 days do not significantly alter the mechanical properties of the specimens [73]. In the group where resin infiltration was performed before bleaching, the interval between resin infiltration and bleaching was 1 day [73]. Conversely, in the group receiving resin infiltration after bleaching, the procedure was postponed for 14 days following bleaching to counteract the inhibitory effect of peroxide on resin polymerization [73].

This study’s investigation into chromatic changes in WSLs under different sequences of resin infiltration and bleaching has shed light on effective treatment protocols, a topic not thoroughly explored in prior research. While Yeslam HE et al. [33] supported resin infiltration prior to bleaching in vitro and Christoph M. Schoppmeier et al. [24] recommended post-bleaching infiltration in clinical settings, Al-Shaheen Youssef et al. [28] found no significant chromatic differences between these sequences, questioning the assumption of sequence-dependent effectiveness. In contrast, our results demonstrate that both pre- and post-bleaching resin infiltration significantly improve immediate color matching, as evidenced by reduced deviation of L*, a* and b* values compared to baseline. However, the longevity of aesthetic results is often undermined by factors such as water absorption by TEDGMA, shrinkage due to polymerization in low-filler resins, and external conditions like thermal and pH cycling, which can deteriorate both resin and enamel. This leads to potential issues like discoloration, plaque build-up, and secondary caries [1, 74]. To evaluate color stability, oral conditions were simulated using thermocycling and staining methods. Post-thermocycling analyses revealed no notable chromatic changes; however, only specimens from the post-bleaching resin infiltration group maintained long-term color stability after staining, which could be attributed to their lower surface roughness [50]. Clinically, to enhance durability, polishing procedures and oral hygiene instruction (OHI) are advised [71].

The outcomes of the present study showed that resin infiltration alone and bleaching prior to resin infiltration significantly increased the surface microhardness of WSLs compared with bleaching after resin infiltration. As ICON treatment progresses during the finishing procedure, there is an increase in surface microhardness. This increase can be attributed to the relatively uniform formation of resin-hydroxyapatite complexes, which fill the voids and spaces in the demineralized zone [20]. In contrast, when bleaching is used as a finishing procedure, enamel composition undergoes significant modification due to the removal of calcium and phosphate, as suggested by Ergucu Z et al. [75]. This results in compromised hardness. The current findings also suggest that the microhardness of negatively affected bleached WSLs can be restored by the resin infiltrant, aligning with the in vivo observations of Horuztepe SA et al. [21].

Thus, we partially refuted the first null hypothesis, advocating post-bleaching resin infiltration as an efficacious approach for the aesthetic management of WSLs, particularly for lesions affecting shallow dentine [72].

When WSLs exceed the depth of dentine, incomplete coverage of resin infiltration might occur, yielding insufficient aesthetic outcomes [72, 76]. Lesions of this nature are often only partially hidden, remaining visible to clinical observation [72]. This necessitates more intensive restorative methods [72], such as direct resin composite, indirect ceramic veneers, and complete coverage crowns [77]. Porcelain laminate veneers are particularly favored due to their balance between conservation and aesthetic results, making them a common selection for treating enamel with unsuccessful infiltration in WSLs.

Furthermore, this research evaluated the effect of resin infiltration on the shear bonding strength (SBS) of porcelain discs to determine the clinical applicability of post-infiltration ceramic bonding. Over the past two decades, the interface between dental ceramic and resin cement has undergone extensive testing through various methodologies. Shear bonding tests, which assess the maximum or failure load before rupture [78], have been a mainstay in studies of bonding strength related to WSLs, particularly those focusing on orthodontic adhesives. While Naidu E et al. [79] observed that resin infiltration not only maintained but also improved the SBS of orthodontic resin cements, Montasser MA et al. [80] found no significant change. To the best of our knowledge, the SBS of porcelain adhesives on WSLs pre-treated with ICON has not been previously explored. Our results indicate that resin infiltration does not adversely affect SBS when using the Variolink N bonding system. An increased occurrence of adhesive failure in failure pattern analysis suggests that resin infiltration may actually enhance the bonding characteristics of ceramic bonding agents. Clinically, this means that resin infiltration is unlikely to increase the risk of marginal interface rupture, which could lead to infiltration of oral fluids and subsequent secondary caries [81, 82].

We also investigated the success and failure of ceramic restoration by examining microleakage at the enamel-ceramic interface post-thermocycling utilizing the dye penetration method with 2% methylene blue dye [83]. Previous studies have suggested that methylene blue penetrates the deepest layer in WSLs (the inner half of enamel or outer half of dentin), secondarily through sound enamel (the outer half of the enamel), and penetrates the most shallow layer in infiltrant-treated WLSs (no penetration or the inner half of enamel) [84]. Like Klaisiri A et al. [84] and Lee J et al. [85], we found that dye penetration depth was reduced more than tenfold in resin-infiltrated WSLs compared to that in WSLs. However, unlike in previous studies, we found no significant difference in microleakage between sound enamel and resin-infiltrated WSLs [84]. Nevertheless, resin infiltration reversed the deficiencies in the integrity of the hydroxyapatite latticework, which in turn prevented microleakage.

Representative SEM micrographs illustrating the microchanges on the WSL enamel surface after ceramic bonding. Owda R et al.[97] observed the rough surface and enlarged interprismatic space characteristic of enamel in WSLs. Our study demonstrated a shift from this rough, demineralized enamel to a more uniform, resin-infiltrated surface, aligning with findings by Horuztepe SA et al. [86]. The improvement in enamel margin continuity with ICON and Syntac adhesives, previously identified by Körner P et al. [87] at ×200 magnification, was further explored in our study at higher magnifications (×2000 and ×5000). A distinct contrast was noted in the WSLs bonding interface, with compromised adhesive polymerization, while the infiltrated WSLs bonding interface showed a seamless transition and uniform integration, indicating enhanced bonding efficiency and potential for better clinical outcomes.

Therefore, the second null hypothesis was partially rejected. Our findings suggest that resin infiltration does not adversely affect bonding integrity and is effective in reducing microleakage in WSLs.

This study highlights the combined benefits of various treatments with resin infiltration for managing WSLs. However, it is important to acknowledge certain limitations. The use of bovine teeth, despite their structural resemblance to human teeth, might result in faster acid and resin penetration [88] than in human enamel. This is due to their larger crystal size, smaller prism diameters, more interprismatic substance, and increased porosity [89]. Still, qualitative findings in the current study can be directly applied in human conditions. Additionally, the controlled laboratory setting of this in vitro study does not entirely replicate the complex environment of the oral cavity. Consequently, in-vivo experiments are necessary to validate the effectiveness of these combined treatment approaches for WSLs.

## Conclusions

Within the limitations of in vitro study, the following conclusions can be drawn:

Post-bleaching resin infiltration proved to be advantageous in the aesthetic treatment of WSLs, leading to greater brightness and lower yellowness both in short-term and long-term. Furthermore, it did not compromise the bond strength of ceramics to WSLs but did reduce the microleakage depth and enhance marginal sealing.

Based on these findings, the sequenced treatment of post-bleaching resin infiltration is recommended, and resin infiltration before ceramic bonding is deemed viable in clinical practice.

### Electronic supplementary material

Below is the link to the electronic supplementary material.

Supplementary Material 1

## Data Availability

No datasets were generated or analysed during the current study.

## Abbreviations

WSLs: White Spot Lesions
SBS: shear bonding strength
