# Supplementary material for: Aesthetic impact of resin infiltration and its mechanical effect on ceramic bonding for white spot lesions
Source: BMC Oral Health. 2024 Mar 21;24:365. doi: 10.1186/s12903-024-04011-4 (PMC10958835; doi:10.1186/s12903-024-04011-4)
Supplement: Supplementary file 1 — Supplementary Material 1 [file 12903_2024_4011_MOESM1_ESM.docx]

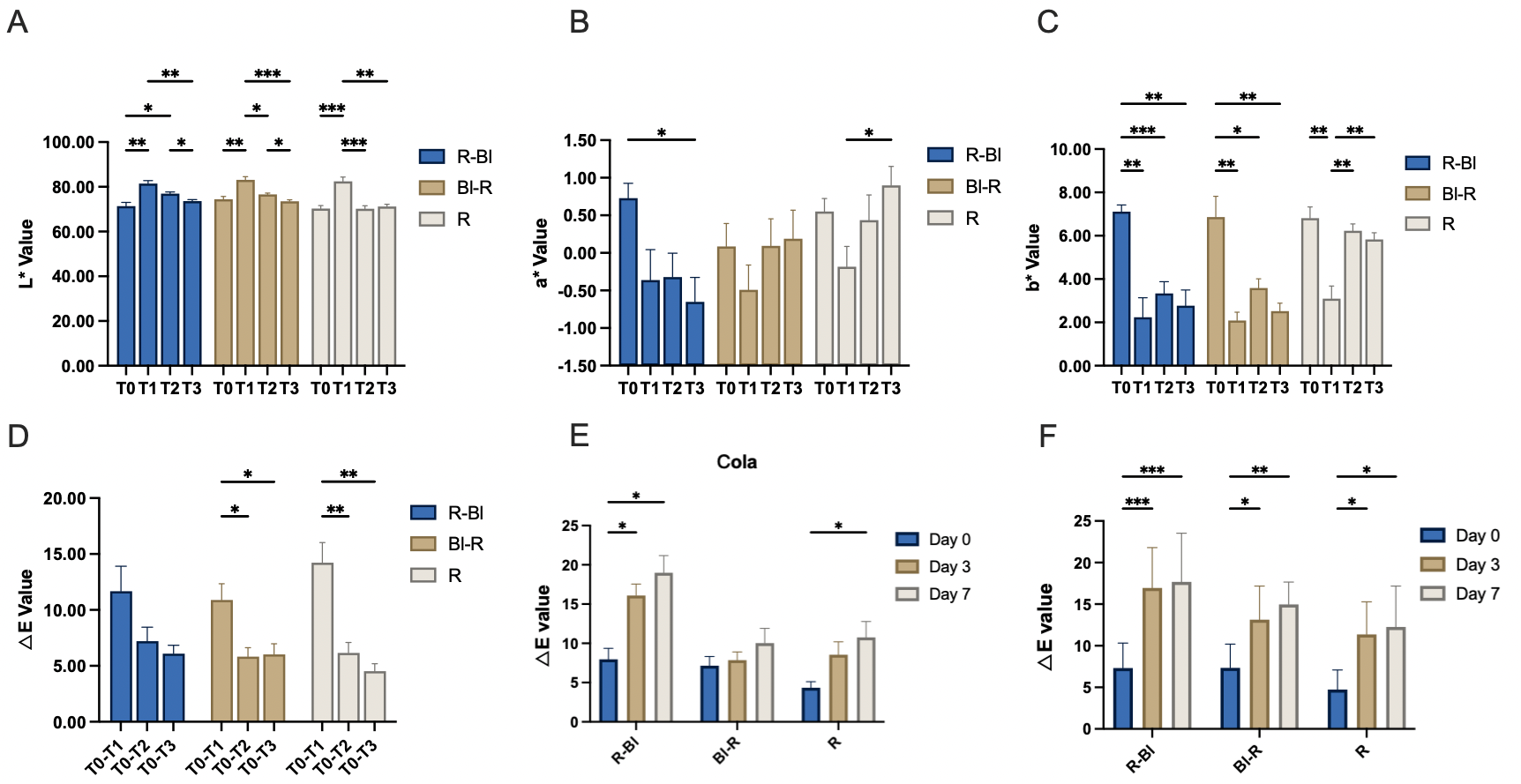


***Fig. S1 The changes in color , surface roughness and microhardness of WSLs treated with resin infiltration and bleaching***

1. The L* values of the R-Bl group, Bl-R group and R group (n=10).

(B) The a* values of the R-Bl group, Bl-R group and R group (n=10).

(C) The b* values of the R-Bl group, Bl-R group and R group (n=10).

(D) The △E values of the R-Bl group, Bl-R group and R group (n=10).

(E) The surface roughness of the R-Bl group, Bl-R group and R group (n=10).

(F) The microhardness of the R-Bl group, Bl-R group and R group (n=10).

T0 (baseline), T1 (after demineralization), T2 (after treatment), T3 (after thermocycling)

* P < 0.033, ** P < 0.002 , *** P < 0.001


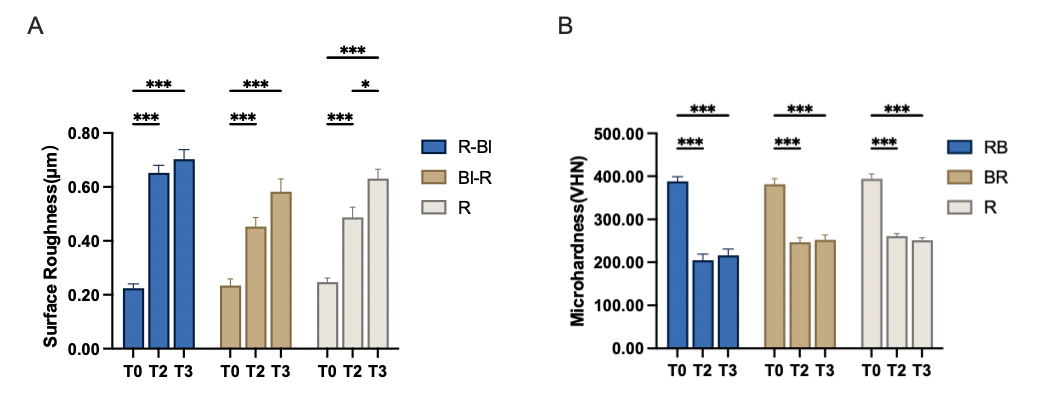


***Fig. S2 The changes in surface roughness and microhardness of WSLs treated with resin infiltration and bleaching***

1. The surface roughness of the R-Bl group, Bl-R group and R group (n=10).

(B) The microhardness of the R-Bl group, Bl-R group and R group (n=10).

T0 (baseline), T1 (after demineralization), T2 (after treatment), T3 (after thermocycling)

* P < 0.033, ** P < 0.002 , *** P < 0.001
